# Granulocyte-Colony Stimulating Factor (G-CSF) Improves Motor Recovery in the Rat Impactor Model for Spinal Cord Injury

**DOI:** 10.1371/journal.pone.0029880

**Published:** 2012-01-12

**Authors:** Tanjew Dittgen, Claudia Pitzer, Christian Plaas, Friederike Kirsch, Gerhard Vogt, Rico Laage, Armin Schneider

**Affiliations:** SYGNIS Bioscience, Heidelberg, Germany; University of North Dakota, United States of America

## Abstract

Granulocyte-colony stimulating factor (G-CSF) improves outcome after experimental SCI by counteracting apoptosis, and enhancing connectivity in the injured spinal cord. Previously we have employed the mouse hemisection SCI model and studied motor function after subcutaneous or transgenic delivery of the protein. To further broaden confidence in animal efficacy data we sought to determine efficacy in a different model and a different species. Here we investigated the effects of G-CSF in Wistar rats using the New York University Impactor. In this model, corroborating our previous data, rats treated subcutaneously with G-CSF over 2 weeks show significant improvement of motor function.

## Introduction

To date there are only very limited treatment options available to improve consequences of spinal cord injuries (SCI) apart from primary surgical intervention for stabilization and decompression and later rehabilitation training. Few pharmacological therapies are currently being evaluated clinically. Given the young age of patients affected by SCI, and the considerable impairment in daily living activities, there is a high need for a treatment that is able to partially restore motor functions to raise the quality of everyday life.

We have shown before that the G-CSF receptor is expressed by neurons in the brain [1] and that G-CSF induces several pathways in neurons to protect against apoptotic cell death. Systemically applied G-CSF passes the intact blood–brain barrier [1], [2]. In stroke models in rodents G-CSF decreases infarct size, and improves functional recovery. Furthermore there is evidence for therapeutic activity in chronic neurodegenerative diseases [3]–[5]. We could recently demonstrate efficacy of G-CSF in a mouse model for spinal cord injury using both subcutaneous and transgenic delivery of the protein [6].

In order to broaden confidence in efficacy of G-CSF in experimental SCI models, we aimed to confirm experimental data gained in one animal species in a second one. In addition, choosing a different experimental disease model should help to uncover false positive treatment effects based on specific characteristics of one model. Previously, we observed improvement of motor function in a model of partial transection of the spinal cord after treatment with G-CSF in mice. In this study we used Wistar rats and an experimental SCI where trauma is induced by the New York University (NYU) Impactor device. Instead of directly transsecting the spinal cord, the tissue is damaged by a weight drop. The impact of the weight onto the surface of the spinal cord leaves the dura intact and leads to an acceleration of the spinal cord tissue comparable to the situation in an accident were the spinal column is rapidly bent. These strong forces act on the long projecting fibres in the spinal cord with the potential consequence of rupture. The impactor model is viewed by many as the best SCI model since it mimicks events occurring during trauma in the human.

## Results

### Conduction of the model

Ten animals received only the laminectomy but no impact (surgery control; n = 10). 51 animals were subjected to the weight drop model using the NYU impactor [7], [8]. This was done to ensure that the paralysis seen in the impactor-injury-group was not the result of the surgery procedure itself. All sham-operated animals showed no sign of motor impairment. 2 animals were excluded from the study due to irregular impact curves. This left 24 animals treated with G-CSF, and 25 with vehicle.

The mean compression rate, calculated by dividing the compression depth by the difference in time between initial spinal cord contact and reversal of the rod (deepest dipping point of the impactor rod), was 0.575±0.0051 m/s in the untreated group and 0.587±0.0057 m/s in the treated group. There was a trend for a higher compression rate in the treated group, the difference between the groups was however not significant (ttest p = 0.12). The compression rate is a measure for the acceleration of the tissue through the impact which leads to the rupture of long projecting fibers. The higher the compression rate, the more the tissue is damaged.

The duration of the surgery before the weight drop injury is an important source of variation in this model. The main reason for delays during surgery is difffuse bleeding following laminectomy that has to be stopped before the impact is delivered. The mean time until impact (mean = 64.62±0.80 min for the untreated group, 64.60±0.86 min for the treated group) was identical for the two groups (ttest p = 0.99). Treatment started immediately after injury with an IV-bolus of 60 µg G-CSF or vehicle per kg bodyweight via a jugular catheter. Subcutaneous minipumps were implanted dorsally and continuously released 30 µg G-CSF/kg bodyweight/day or vehicle over 2 weeks. This treatment scheme was chosen because of the limited plasma half-life of G-CSF (∼4 h).

### Outcome analysis

Six animals died before the observation period was completed, three in the G-CSF group and three in the vehicle group. This left n = 21 animals in the G-CSF group, and n = 22 animals in the vehicle group for which data until day 35 were available. The primary pre-specified analysis of the data includes all animals (G-CSF: n = 24; vehicle: n = 25), and uses the standard procedure last-observation-carried-forward (LOCF) for replacement of missing values.

**Figure 1:**
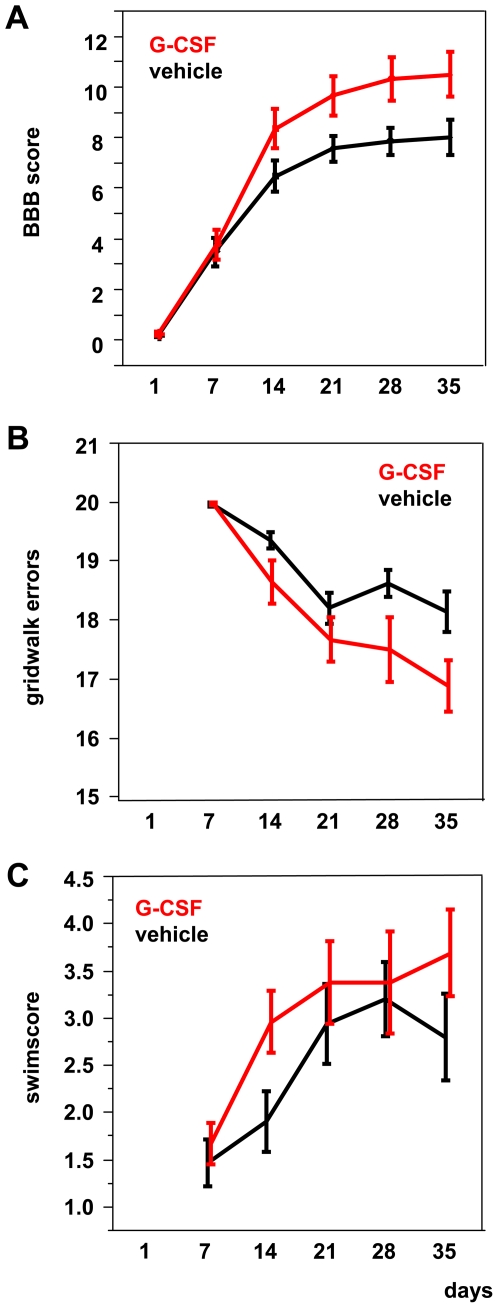
Outcome analysis in rats subjected to weight drop. (**A**) Mean BBB scores in untreated (−) and G-CSF-treated (+) rats from 1 to 35 days, including all data (LOCF) (n = 24 for G-CSF treatment, n = 25 for vehicle treatment; p<0.05 for factor *treatment*, p<0.005 for interaction treatment*time (days after injury) by linear regression analysis). (**B**) Mean Grid Walk scores in untreated (−) and G-CSF-treated (+) rats from 7 to 35 days, including all data (LOCF) (p<0.05 for factor *treatment*, p<0.005 for interaction treatment*time (days after injury) by linear regression analysis). (**C**) Mean Swim Test scores in untreated (−) and G-CSF-treated (+) rats after 7 to 35 days, including all data (LOCF). Data is represented as mean ± SEM. This test.

The Basso-Beattie-Bresnahan score (BBB score) rates the motor abilities of the hindlimbs from 0 = no movement to 21 = normal movement [9]. A BBB score of 9 and above indicates weight supported steps. The mean BBB scores in the untreated group were 0.19±0.06 (day 1), 3.48±0.56 (day 7), 6.48±0.62 (day 14), 7.56±0.52 (day 21), 7.87±0.55 (day 28) and 8.00±0.70 (day 35) compared to 0.26±0.09 (day 1), 3.78±0.58 (day 7), 8.35±0.79 (day 14), 9.66±0.77 (day 21), 10.31±0.88 (day 28) and 10.50±0.90 (day 35) in the treated group (**Figure 1A**). There is a significant difference between the two groups with a positive influence of G-CSF treatment on the motor behaviour of the animals (p<0.05 for factor *treatment*, p<0.005 for interaction treatment*time (days after injury) by linear regression analysis). Omitting animals that died before the end of the test series and removing all imputed (LOCF) values did not change this effect.

The Grid Walk test requires advanced motor abilities and animals with less than 16 errors are already well recovered. We observed a significant difference between the two groups with a positive influence of G-CSF treatment on the motor behaviour of the animals (**Figure 1B**; p<0.05 for factor *treatment*, p<0.005 for interaction treatment*time (days after injury) by linear regression analysis). Again, omitting dead animals and removing LOCF values had no considerable effect on interpretation of the outcome.

In the Swim Test the animals are moving in a narrow water basin of 1 m length. A ladder at the opposite end motivates the animals to cross the basin. The hindlegs are analyzed and rated from 0 (no movement) to 10 (normal movement). Although there is a visible trend towards better performance (**Figure 1C**) this difference failed significance.

## Discussion

This study aimed to confirm evidence for efficacy of G-CSF in the treatment of SCI originally obtained in a mouse transection model in a different species with a different injury model [6]. The weight drop model in rats showed clearly beneficial effects of G-CSF treatment for the main motor outcome parameters BBB and Grid Walk. No significant change between groups could be detected for the Swim Score, a test that certainly has less statistical power in SCI models as also observed previously [6]. Post-hoc analyses of our data revealed a power of only 18% to detect a 25% difference in means.

Here, we demonstrated for the first time the beneficial effects of G-CSF in a spinal cord contusion model in the rat using the NYU impactor. The spinal cord injury model with the NYU impactor appears currently as the best contusion model available [8], and has been judged as very similar to the pathophysiology of human spinal cord contusion injuries in a systematic comparison [7]. The induced motor deficit in the impactor model is also more severe than injury induced by partial spinal cord dissection, and the amount of recovery does not seem as big here as in our previous studies [6]. In the absence of any successful SCI therapy in man it is always difficult to judge the meaning of an effect size in animal models. Although the grid walk score appears as a highly continuous linear scale this score suffers from a lower border ceiling effect lacking discriminatory sensitivity (all animals after SCI models start out with 20 errors), and a limited factual range: To reach a value of 16 errors the animals have to be already well recovered. The true range of this score is therefore far more limited than a true linear range of 0–20. In the important functional BBB score there is a relative increase in performance by 25%, which appears substantial also with regard to possible translations of this therapy to humans.

A number of other labs has performed spinal cord injury and ischemia models with G-CSF treatment alone, or in combination or control group with some sort of cellular transplantation therapy [6], [10]–[15]. **Table 1** lists all published data on G-CSF treatment approaches in SCI models. All studies have been performed in either mice or rats. Two groups have used compression models, two groups used transection models, static contusion was used by one group, and one group has employed an ischemic model. Treatment duration was between one and 14 days. Drug application modes were subcutaneous, intrathecal, or intravenous. Doses given ranged from 50 µg/kg bodyweight up to 300 µg/kg bodyweight, with one group using 10 µg G-CSF for intrathecal injections without bodyweight adjustment. All groups report functional outcome in the animals, with 5 publications reporting the BBB score. Two groups used combination therapy with stem cells (neural stem cells [14] or bone marrow derived cells [10]), one group used a combination with stem cell factor [15], the four other publications use G-CSF only [6], [11]–[13], Urdzikova et al. use a G-CSF only control group in addition to bone marrow stem cell combination [10].

**Table 1:** Listed are the different spinal cord injury models in rodents where G-CSF demonstrated beneficial effects.

SCI models	Species	Application mode and treatment duration	Outcomes and behavioural testing	References
Balloon induced compression	rat	+/− combination bone marrow stem cells and i.v. 50 µg/kg bodyweight/day; 5 days	+functional outcome; BBB and plantar test	Urdzikova et al., 2006
Compression	mice	s.c. 200 µg/kg bodyweight/day; 5 days	+functional outcome;motor function scale	Nishio et. al, 2007
Spinal cord ischemia	rat	i.t. 10 µg; 1 day	+functional outcome; MDI	Chen et al., 2008
Spinal cord ischemia	rat	i.t.10 µg; 1day	+functional outcome, BBB	Chen et al., 2010
Transection	rat	Combined NSC+ s.c. G-CSF 50 µg/kg bodyweight/day; 5 days	+functional outcome; BBB	Pan et al., 2008
Static contusion	mice	Combined SCF and s.c. G-CSF 300 µg/kg bodyweight/day; 10days	+functional outcome, BBB LRS	Osada et al., 2010
Transection	mice	i.v. 60 µg/kg bodyweight; 1day and s.c. 30 µg/kg bodyweight; 14 days	+functional outcome; BBB, swim test and grip walk	Pitzer et al., 2010

Given are the respective spinal cord injury models, the animal species, the application mode and the outcome of the experiment with the behavioural testings used (+: significant benefit; s.c. subcutaneously; i.v. intravenously; i.t. intrathecally; NSC: neural stem cells; SCF: Stem Cell Factor; MDI: Motor Deficit Index; BBB: Basso, Beattie and Bresnahan locomotor score).

The mechanisms that are discussed as being responsible for the beneficial effect of G-CSF are induced proliferation of oligodendroglial precursors [15], inhibition of apoptotic cell death via activation of protective pathways (Akt, ERK, stat3 – Bcl2 - Bcl-Xl) [6], [11], [12], suppression of excitotoxicity [13], support for stem cells [10], [14], and an influence on neuronal plasticity and spinal cord tract connectivity [6]. This multimodal activity of G-CSF indeed is a principal argument for increased success probability in the clinic as opposed to singular approaches as has also been discussed in the stroke field [16].

All SCI studies cited above report improved functional recovery following G-CSF therapy. We are the first here to report functional improvement after G-CSF treatment in the impactor model, often regarded as the likely most relevant model for traumatic spinal cord injury. Together these are 8 reports on activity of this growth factor in various models, and from different labs. Together with the data from our lab this forms a credible body of evidence that G-CSF has a robust effect in SCI in different models and species. High doses of G-CSF appear safe in acute neurological disease in man [17]. G-CSF is therefore a candidate drug for spinal cord injury therapy that can be rapidly translated into the clinic.

## Materials and Methods

### Ethics statement

All animal experiments were conducted in agreement with national and international guidelines. Care was taken to minimize suffering for the animals. Over the entire observation period animals received Tramadol (Tramal-ratiopharm® (Ratiopharm GmbH), added to drinking water, 15 mg/kg BW/d) in the drinking water for pain management. Animal experiments were approved by the Regierungspräsidium Karlsruhe (AZ 35-9185.81/G-15/05).

### Experimental SCI by weight drop

Wistar rats were provided by Charles River Laboratories. Acclimatization was done for 1 week before starting the study. Animals were housed using standard procedures with normal light-dark periods. Special care was taken to allow adequate access to food and water during the trial. Female rats of ∼300 g (app. 2 months of age) were anesthetized by inhalation of 70% N_2_O, 30% O_2_ and 1% Halothane. Laminectomy was performed at spinal level T9/10 followed by an impact of a 10 g metal rod from 25 mm height onto the exposed spinal cord with intact dura. The weight drop was performed with the help of the NYU (MASCIS) Impactor directly obtained from Dr. Wise Young, Rutgers University, New York. Every impact and the resulting forces on the tissue were monitored and documented on-line to control for uniform severity of the injury. The animals were monitored daily for signs of stress, pain, decubitus, autophagia, autotomia, or dehydration. The bladder was emptied manually twice per day (Credé maneuver); the first week after the surgery animals received Cotrimoxazol (Cotrim-ratiopharm® (Ratiopharm GmbH), solution for IP-injection 50 mg/kg BW/d, diluted in Ringer)) to prevent urinary tract infections. Over the entire observation period animals received Tramadol (Tramal-ratiopharm® (Ratiopharm GmbH), added to drinking water, 15 mg/kg BW/d) in the drinking water for pain management.

### Treatment

The treatment started immediately after injury with an IV-bolus of 60 µg G-CSF (Filgrastim 30 MU/ml) or vehicle/kg BW via a jugular catheter. Vehicle and the G-CSF buffer solution consisted of 10 mmol/L HAc/NaAc, pH 4.0, 250 mmol/L Sorbitol, 0.004% (v/v) Tween-80. In addition, an osmotic minipump (Alzet 2002) was implanted subcutaneously delivering 30 µg/kg G-CSF BW/day for 14 days or vehicle.

### Behavioural assessment

The animals' motor behavior was monitored weekly for 5 weeks postinjury. All behavioral tests were recorded with a video camera and scored independently by two observers blinded to the treatment.

#### BBB locomotor score

The general locomotor performance of the animals was assessed using the BBB locomotor rating scale with slight modifications [9]. In brief, rats were placed on a runway of 1.2 m length and 10 cm width on top of an inclined mirror (60° angle) to facilitate the observation of hind-limb movements. Hindlimb locomotion was scored from 0 (for no observable hindlimb movement) to 21 points (regular movement). Every animal had to cross the runway three times. The BBB test was performed in weekly intervals and at day 1 after SCI.

#### Grid walk test

Deficits in descending motor control were examined by assessing the ability to navigate across a 1.2 m long runway with irregularly spaced metal bars and gaps of 0.5–2 cm [18]. A defined 10-bar-sector was chosen for the analysis. To prevent habituation to a fixed bar distance, the bars in this sector were changed for each testing session. The performance was scored by counting the number of errors in foot placement per 20 steps (10 for each foot). If the animal was unable to use its hindlimbs for weight support, it made two errors per bar resulting in a total of 20 errors. When the hindlimb paw was not accurately placed on the bar, this was counted as error. Any misplacement of the paw, any slippage between rungs was counted as error. To perform well on this test animals must show advanced motor-sensory abilities, and have to have weight supported. Spastic movements of the hindlimb are not considered an improvement and can be clearly distinguished from correct plantar placement. Uninjured control animals usually made no or very few mistakes. The grid walk test was performed once a week for 5 weeks after SCI.

#### Swimming score

A swimming test was used to determine the locomotor performance of injured rats in absence of cutaneous and proprioceptive sensory input from the hindlimbs. The animals had to swim in a 1.2 m long and 10 cm wide water tank. Swimming performance was evaluated by scoring the following features: hindlimb movement, hindlimb-forelimb coordination, tail position, paw position and lateral stability. Uninjured rats received 10 points, while completely paraplegic animals scored 0 points as previously described [19]. A detailed scoring protocol is available as **Table S1**. Each rat had to cross the tank twice. The swimming test was performed weekly for 5 weeks after SCI.

### Statistics

All experiments were conducted in a completely randomized and blinded fashion using computer-generated probe allocation, and separation of experiment conduction from data analyses. Statistical analyses were done using JMP 8.01 (SAS Institute). Data analyses were performed as closely as possible to clinical trial standards (adhering to the International Conference on Harmonization (ICH) E9 guideline where applicable), entering all animals that had received treatment and where at least one behavioural data point was available into the analysis according to the Full Analysis Set (FAS) definition. If an animal did not survive the full observation period, the last observed score was carried forward (LOCF). In secondary analyses we also performed all analyses with omission of missing values. For comparison of two groups, t-test statistics was used. For time series of measurements linear regression analysis was performed using time, treatment, the interaction of both as factors, and animal identity as random effect. P-values of <0.05 were regarded as statistically significant. Values are given as mean±SEM.

## Supporting Information

Table S1 **Swimming Score.** The swimming performance of the rat was evaluated by scoring the following features: hindlimb movements, hindlimb-forelimb coordination, tail position, paw position, sagittal and coronal balance. Scoring scales are added and maximal reached scale is 10.
(PDF)
