# Supplementary material for: Granulocyte-Colony Stimulating Factor (G-CSF) Improves Motor Recovery in the Rat Impactor Model for Spinal Cord Injury
Source: PLoS One. 2012 Jan 12;7(1):e29880. doi: 10.1371/journal.pone.0029880 (PMC3257226; doi:10.1371/journal.pone.0029880)
Supplement: Table S1 — Swimming Score. The swimming performance of the rat was evaluated by scoring the following features: hindlimb movements, hindlimb-forelimb coordination, tail position, paw position, sagittal and coronal balance. Scoring scales are added and maximal reached scale is 10. (PDF) [file pone.0029880.s001.pdf]

<table>
<tr><th colspan="2">Paw Position</th><th colspan="6">Movement</th><th colspan="3">Hindlimb-Forelimb coordination</th><th colspan="2">Sagittal balance</th><th colspan="2">Tail</th></tr>
<tr><td rowspan="4">90° to trunk</td><td rowspan="4">Parallel to trunk</td><td rowspan="4">no</td><td colspan="2">one joint</td><td colspan="3">> one joint</td><td rowspan="4">no</td><td colspan="2">yes</td><td rowspan="4">no</td><td rowspan="4">yes</td><td rowspan="4">down</td><td rowspan="4">up</td></tr>
<tr><td rowspan="3">slight</td><td rowspan="3">extr.</td><td rowspan="3">slight</td><td colspan="2">extr.</td><td rowspan="3">occass.</td><td rowspan="3">consist.</td></tr>
<tr><td colspan="2">Cor.balance</td></tr>
<tr><td>no</td><td>yes</td></tr>
<tr><td>0</td><td>1</td><td>0</td><td>1</td><td>2</td><td>3</td><td>4</td><td>5</td><td>0</td><td>1</td><td>2</td><td>0</td><td>1</td><td>0</td><td>1</td></tr>
</table>
